# Cross-cultural adaptation and psychometric analysis of Teachers’ Self-Efficacy in Asthma Management

**DOI:** 10.1590/0034-7167-2025-0438

**Published:** 2026-09-21

**Authors:** Francisco Figueiredo, Bruna Josiane de Lima, Ingrid Daiane Silva Aiello, Aline Helena Appoloni Eduardo, Edmara Bazoni Soares Maia, Aline Cristiane Cavicchioli Okido

**Affiliations:** I Universidade Federal de São Carlos. São Carlos, São Paulo, Brazil; II Universidade Federal de São Paulo. São Paulo, São Paulo, Brazil; III Universidade de São Paulo. Ribeirão Preto, São Paulo, Brazil

**Keywords:** Validation Study, Self Efficacy, Asthma, Child, School Teachers., Estudio de Validación, Autoeficacia, Asma, Niño, Maestros.

## Abstract

**Objective:**

to perform cross-cultural adaptation and psychometric analysis of Teachers’ Self-Efficacy in Asthma Management for Brazil.

**Methods:**

a psychometric study encompassing the stages of translation, translation synthesis, back-translation, expert committee submission, pre-final version testing, and adapted version testing. Confirmatory factor analysis and Cronbach’s alpha coefficient were performed to analyze construct validity and reliability.

**Results:**

the Content Validity Index of the translated version was higher than 0.80, after modifications proposed by experts. Pre-final version testing indicated good comprehension and ease of response. The adapted version was tested on 143 teachers. Confirmatory factor analysis demonstrated a good fit to the model, with factor loadings between 0.555 and 0.911. Cronbach’s alpha coefficient was 0.922.

**Conclusion:**

the Brazilian version of the scale presented reliable and valid psychometric measures, confirming its unidimensionality.

## RESUMEN

**Objetivo:**

realizar la adaptación transcultural y el análisis psicométrico de la escala Teachers’ Self-Efficacy in Asthma Management para el Brasil.

**Métodos:**

estudio psicométrico que abarcó las etapas de traducción, síntesis de las traducciones, retrotraducción, presentación a un comité de expertos, evaluación de la versión prefinal y evaluación de la versión adaptada. Se realizó un análisis factorial confirmatorio y el coeficiente alfa de Cronbach para analizar la validez de constructo y la fiabilidad.

**Resultados:**

el Índice de Validación de Contenido de la versión traducida fue superior a 0,80, tras las modificaciones propuestas por los expertos. La prueba de la versión prefinal indicó buena comprensión y facilidad de respuesta. La versión adaptada se evaluó con 143 docentes. El análisis factorial confirmatorio demostró un buen ajuste al modelo, con cargas factoriales entre 0,555 y 0,911. El coeficiente alfa de Cronbach fue de 0,922.

**Conclusión:**

la versión brasileña de la escala presentó medidas psicométricas fiables y válidas, lo que confirma su unidimensionalidad.

## INTRODUCTION

Asthma is among the most prevalent chronic diseases in the child population, affecting more than 300 million people worldwide^(1)^. In the United States, it is estimated that currently 4.6 million children and adolescents have this diagnosis^(2)^. In Brazil, a study conducted with 16,237 children treated at an outpatient clinic of a reference pediatric hospital identified asthma as the most frequent diagnosis among the cases analyzed^(3)^.

Asthma management’s main goal is to reduce the risk of exacerbations and improve individuals’ quality of life^(4)^. Among the non-pharmacological measures that contribute to managing symptoms and improving quality of life in asthmatic children, the following stand out: avoiding exposure to tobacco, reducing contact with polluted environments, promoting weight loss in overweight children, and encouraging the practice of breathing exercises^(5)^. Pharmacological management, in turn, should be guided according to the clinical severity and level of disease management. Despite the availability of effective treatments, uncontrolled asthma remains highly prevalent, frequently requiring hospital care. In this regard, according to data from the Brazilian Health System Department of Information and Informatics, known as DATASUS, in 2019, Brazil registered 53,352 hospitalizations of children between 0 and 14 years old due to asthma, with 8,074 in the state of São Paulo alone^(6)^.

Considering that asthma is prevalent in the 5-14 age group, that these children spend, on average, one-third of the day in school settings, and that most schools do not have nursing professionals present, the literature highlights the importance of the role of teachers in promoting child well-being in the classroom. Furthermore, it emphasizes the need for teachers to recognize signs of uncontrolled asthma, since exposure to triggers throughout school day can trigger severe and potentially fatal attacks^(4)^. However, the absence of specific training and the lack of adequate knowledge to deal with asthma-related emergencies generate discomfort and insecurity among teachers^(7)^.

Managing a child with asthma symptoms can be an extremely challenging and distressing experience, especially in the presence of knowledge gaps. This condition negatively impacts teachers’ self-efficacy, compromising their ability to act quickly and assertively in the face of the situation^(8)^. The concept of self-efficacy, introduced by Bandura in 1977, refers to an individual’s confidence in their own ability to successfully perform a given action and achieve the desired results^(9)^.

In the United States, approximately 45% of schools have a full-time nurse^(10)^. In Brazil, the presence of a dedicated nursing professional is observed only in some private educational institutions. It is in this context that the Health at School Program is situated, a Brazilian public policy created to meet local demands through intersectoral articulation between the education sector and Family Health Strategy units. However, the program’s problem-solving potential has presented significant limitations that hinder its expansion and effectiveness. Among the main challenges are gaps in professional training, weaknesses in information monitoring, lack of coordinated planning among teams, communication failures, insufficient infrastructure, and insufficient public investment^(11)^.

Following this perspective, American researchers developed a scale entitled Teachers’ Self-Efficacy in Asthma Management. The instrument consists of 12 items that assess teachers’ self-efficacy in different dimensions of asthma management, including symptom recognition, trigger prevention, medication administration, communication with parents, encouragement of participation in physical activities, and management of emergency situations. The scale uses a five-point Likert scale, with responses ranging from 1 (completely insecure) to 5 (completely confident). The final score is obtained by summing the scores assigned to each item, and can range from 12 to 60 points. The higher the score obtained, the greater the level of self-efficacy perceived by teachers^(7)^.

Given the above, and considering the scarcity of studies addressing teachers’ self-efficacy in asthma management, the originality of this study is justified by proposing the cross-cultural adaptation and psychometric analysis of Teachers’ Self-Efficacy in Asthma Management, since there is currently no equivalent instrument validated for the Brazilian context. Furthermore, the social relevance of the proposal is highlighted, as it provides a tool with the potential to support the planning and implementation of systematized and effective educational strategies aimed at teachers, contributing to the care and safety of children with asthma in school settings.

## OBJECTIVE

To perform cross-cultural adaptation and psychometric analysis of Teachers’ Self-Efficacy in Asthma Management for Brazil.

## METHODS

### Ethical aspects

The research project was submitted to and approved by a Research Ethics Committee, in accordance with the ethical principles established by Resolution 510/2016 of the Brazilian National Health Council. It should also be noted that prior contact was made with the researcher responsible for developing the original instrument, who formally authorized the conduct of this study in Brazil.

### Study design, period and place

This is a psychometric study that went through the stages of initial translation, translation synthesis, back-translation, expert committee submission, pre-final version testing, and adapted version testing^(12)^. The entire methodological process was carried out between March 2022 and August 2024. The first four stages were conducted through electronic communication between researchers, translators, and expert committee members. The pre-final version was tested in public and private early childhood, elementary, and high schools in a municipality in the countryside of the state of São Paulo. The application of the adapted version was conducted remotely, without geographical limitations for data collection, which allowed the participation of teachers from different locations in the country.

### Population and sample: inclusion and exclusion criteria

The translation, synthesis, and back-translation stages involved two independent Brazilian translators fluent in both languages. As for the expert committee, inclusion criteria encompassed professionals with expertise in pediatric asthma, with academic and/or clinical-assistance experience, as well as researchers with experience in cultural adaptation studies. Eligibility criteria for the fifth and sixth stages included teachers over 18 years of age with at least one year of experience in public or private schools. No exclusion criteria were established.

### Data collection and organization

#### Initial translation, translation synthesis, and back-translation

Initially, the scale was translated into Portuguese by two independent translators. Then, the research group met to analyze the two versions produced and, by consensus, develop a translation synthesis. The resulting synthesis was then back-translated into English in order to be compared with the original version of the instrument.

#### Expert committee submission

Experts were recruited through consultation of the *Lattes* platform and using a snowball sampling strategy^(13)^. Eligible professionals received an invitation to participate in the research via email, containing a link that directed them to Google Forms^®^. The Informed Consent Form (ICF) was available on the form’s homepage, and only after expressing their agreement did experts have access to the consensual version of the scale for assessment.

The expert committee assessed the translated version considering the conceptual, operational, and semantic equivalences of items, using a Likert scale with the following response options: -1 (disagree), 0 (undecided), and 1 (agree). Participants were instructed to comment and suggest improvements whenever they assigned the responses -1 or 0. Furthermore, they answered questions to characterize their profile, such as area of study, age, years since graduation, qualifications, and area of professional activity. The estimated time for analysis and completion of the form was approximately 30 minutes.

#### Pre-final version testing

This stage aimed to verify the instrument’s acceptability and understanding by the target audience, i.e., the teachers. A proper understanding of the items is fundamental to avoid confusion regarding the overall meaning of the scale and errors in the interpretation of specific terms or concepts.

To this end, contact was established with directors and/or coordinators of public and private early childhood, elementary, and high schools in the municipality where the research was conducted, in order to present the project and request authorization for its development. After obtaining authorization, the researchers invited potential participants in person. Those who agreed to participate answered a sociodemographic questionnaire (sex, age, years since graduation, presence of an asthma diagnosis or family history of asthma), in addition to answering the version of the scale validated by an expert committee.

Subsequently, participants conducted a general analysis of the scale, indicating whether items were clear and understandable, as well as whether they encountered any difficulties in responding. There was room for suggestions and comments. Finally, teachers were invited to specifically analyze four items of the scale. For this, the 12 items were randomly divided into three subsets of four items each, and participants were distributed among these groups. This analysis was conducted through recorded interviews, in which teachers were asked to rephrase each item in their own words. This approach allowed researchers to assess participants’ level of understanding and identify terms or expressions that could contribute to greater clarity of the instrument. This strategy was based on DISABKIDS^®^ recommendations and instruments^(14)^.

#### Adapted version testing

Initially, a poster was created containing the study’s objective and an invitation addressed to teachers. The poster was disseminated through email, sharing on social media (such as WhatsApp^®^ and Facebook^®^ groups), and posting on bulletin boards in schools in the researcher’s home municipality. Teachers interested in participating accessed the research form via a QR code or link provided on the poster itself.

Upon completing the form, participants first had access to the ICF. Only after agreeing to the terms they began filling out the scale, along with a characterization questionnaire, which included information such as age, years of experience, area of expertise, and type of educational institution. All teachers who accessed the ICF agreed to participate in the research, with no refusals recorded. The estimated mean time to complete the instrument was approximately five minutes.

### Data analysis

To analyze data relating to the fourth stage (expert committee submission), evaluators’ suggestions were considered, calculating the Content Validity Index (CVI) per item in each of the aspects analyzed, such as conceptual equivalence, item equivalence, operational equivalence, and semantic equivalence. For content validity purposes, a CVI equal to or greater than 0.80 was considered adequate^(15)^.

To examine the psychometric properties of the adapted version, only a confirmatory factor analysis (CFA) model was used, since the objective was to test the unidimensional structure previously defined in the original instrument; therefore, exploratory factor analysis was not performed. Factor extraction followed the Weighted Least Squares Mean and Variance Adjusted estimator, appropriate for ordinal items and based on a polychoric correlation matrix. Regarding the models tested, only one confirmatory model was estimated, corresponding to the unifactorial structure originally proposed for the instrument, in order to assess the adequacy of the theoretical model to the studied sample.

To assess the fit of the models to the data, the following indices were used: chi-square (χ^2^); Comparative Fit Index (CFI); Tucker-Lewis Index (TLI); Root Mean Square Error of Approximation (RMSEA); and Weighted Root Mean Square Residual (WRMR). Values of CFI and TLI ≥ 0.90^(16)^, RMSEA ≤ 0.06^(17)^ and WRMR < 1.0^(18)^ were considered indicative of a good fit. The residual polychoric correlation matrix was analyzed to identify possible underestimations or overestimations of the relationships among variables, with residuals between -0.1 and 0.1 considered acceptable.

After CFA, data were described using absolute and percentage frequencies, for qualitative variables, as well as measures of central tendency and dispersion, such as mean, standard deviation (SD), minimum, median, and maximum, for quantitative variables. Reliability was assessed using internal consistency, employing Cronbach’s alpha coefficient, with a value greater than 0.70 considered satisfactory^(19)^.

The floor/ceiling effects were analyzed based on the percentage of participants who achieved, respectively, the minimum or maximum possible score. A relevant effect is considered to exist when more than 15% of participants achieve one of the extreme scores^(20)^. To compare the total self-efficacy score according to the variables of interest (age range and years of education), a regression model with a negative binomial distribution and logarithmic link function was used^(21)^.

All graphs were created using RStudio version 2023.12.0.369, while statistical analyses were conducted using SAS 9.4, except for CFA, which was performed using MPLUS 6.12. A significance level of 5% was adopted for all analyses. Statistical analyses were supported by a professional in the field.

## RESULTS

The expert committee was composed of 24 medicine (pediatrics), nursing, and physiotherapy professionals. Participants’ mean age was 35.5 years (SD = 7.3), with a mean training time of 10.9 years (SD = 3.5). Regarding academic qualifications, 33.3% held a doctoral degree, 23.8% a master’s degree, and 42.9% were specialists. Concerning professional activity, 28.6% were teachers, and 71.4% worked in healthcare.

The CVI for the “semantic equivalence” criterion was below the established limit in ten items. In the other criteria assessed, all items reached scores above 0.80 in the first round. Among the main suggestions from experts to improve item clarity, the following stand out: replacing “trigger” (*gatilho*) with “factors that trigger asthma” (*fatores que desencadeiam a asma*), “inhaler” (*inalador*) with “inhaler device for asthma (puffer)” (*dispositivo inalatório para asma (bombinha*)), “asthma attack” (*ataque de asma*) with “asthma crisis” *(crise de asma*), and “blue lips” (*lábios azuis*) with “bluish lips” (*lábios arroxeados*). Based on these suggestions, a new version of the scale was developed, incorporating the proposed modifications, and submitted to a second round of assessment, in which consensus was reached among experts.

In pre-final version testing, 30 teachers participated, mostly female, with a mean age of 37.9 years (SD = 9.4) and a mean training time of 11.2 years (SD = 4.1). One teacher reported having a diagnosis of asthma, and 27.3% had a family member with asthma. Table 1 presents the overall assessment of the instrument, showing that most considered the scale “very good” and “easy to understand”. Concerning the response categories, 86.7% indicated that they did not find it difficult, and all considered the questions to be “very important”.

**Table 1:** Distribution of teachers (N = 30) according to response category of the general printout form. São Carlos, São Paulo, Brazil, 2024

Form items	n (%)
**What do you think of our instrument in general?**	
Very good	17 (56.7)
Good	13 (43.3)
Average/So-so	0 (0)
**What did you think of the questions?**	
They were all easy	23 (76.7)
Some were difficult	07 (23.3)
They were all difficult	0 (0)
**Regarding the response categories, did you have any difficulty using them?**	
No difficulties	26(86.7)
Some difficulties	04 (13.3)
Many difficulties	0 (0)
**Are these questions important to the health/illness of your students?**	
Very important	30 (100)
Sometimes important	0 (0)
Not important at all	0 (0)
**Would you like to change anything about the instrument?**	
Yes	2 (6.7)
No	28 (93.3)
**Would you like to add anything to the instrument?**	
Yes	03 (10)
No	27 (90)
**Were there any questions you did not want to answer? If so, why?**	
Yes	01 (3.3)
No	29 (96.7)

In the specific analysis, it was found that, in general, participants were able to adequately explain the items on the scale, indicating a satisfactory level of understanding. From empirical material analysis, the researchers identified the recurrence of certain expressions used by participants, characterized by clearer and more accessible language. Based on this, the following substitutions were made in the final version of the instrument: “how safe” (*quão seguro*) was replaced by “how safe are you” (*o quanto você está seguro*) (in all items); “assess if” (*avaliar se*) by “identify that” (*identificar que*) (in item 8); and “communicate with parents” (*comunicar com os pais*) by “talk to parents” (*conversar com os pais*) (in item 10).

The adapted version was applied to 143 teachers. Of these, 84 (58.7%) were over 40 years of age, and the mean time since graduation was 17.43 years (SD = 10.25), ranging from 1 to 42 years. Regarding their area of work, 26 teachers (18.2%) worked in early childhood education, 28 (19.6%) in elementary school I, nine (6.3%) in elementary school II, 16 (11.2%) in high school, and 64 (44.8%) in more than one educational level. As for the type of institution, 124 participants (86.7%) worked in public schools.

Table 2 presents the values obtained for the fit indices considering a unifactorial model: χ^2^; CFI; TLI; RMSEA; and WRMR. It can be observed that the CFI and TLI values were greater than 0.90, indicating a good model fit. In contrast, the RMSEA value was above the acceptable limit, suggesting an unsatisfactory fit. Finally, the WRMR value in the respecified model was 0.652, indicating an acceptable fit.

**Table 2:** Description of the initial statistical indices. São Carlos, São Paulo, Brazil, 2024

Indices	Initial model	Re-specified model
RMSEA	0.176 (IC90%: 0.156-0.196)	0.090 (IC90%: 0.066-0.114)
CFI	0.943	0.987
TLI	0.931	0.982
χ^2^	291.934 (54)	103.236 (48)
WRMR	1.361	0.652

*Legend: RMSEA - Root Mean Square Error of Approximation; CFI - Comparative Fit Index; TLI - Tucker-Lewis Index; χ^2^ - chi-square; WRMR - Weighted Root Mean Square Residual; 90%CI - 90% Confidence Interval.*

Table 3 below presents the residual polychoric correlation matrix, obtained from the discrepancies between the observed and estimated correlations. It can be seen that the residuals remained in the range of -0.1 to 0.1, indicating no evidence of underestimation or overestimation in the relationships among variables.

**Table 3:** Residual correlation matrix. São Carlos, São Paulo, Brazil, 2024

	Item 1	Item 2	Item 3	Item 4	Item 5	Item 6	Item 7	Item 8	Item 9	Item 10	Item 11
**Item 2**	0.031										
**Item 3**	0.030	0.000									
**Item 4**	-0.009	0.018	0.041								
**Item 5**	-0.018	-0.037	-0.023	-0.017							
**Item 6**	-0.058	0.065	-0.060	-0.015	0.000						
**Item 7**	-0.087	0.021	-0.074	0.007	0.079	0.000					
**Item 8**	-0.007	-0.022	-0.001	-0.093	0.038	0.007	-0.050				
**Item 9**	-0.062	0.015	-0.095	-0.030	-0.064	0.124	0.126	-0.008			
**Item 10**	0.032	-0.070	0.006	-0.009	-0.027	-0.060	-0.052	0.040	0.000		
**Item 11**	-0.006	-0.019	0.063	0.052	-0.020	-0.062	-0.107	0.030	-0.144	0.048	
**Item 12**	-0.042	-0.021	-0.021	0.089	-0.083	0.062	0.077	-0.062	0.049	0	0

Subsequently, factor loadings were calculated (Table 4), which indicate the correlation of each variable with the factor. It can be observed that all factor loadings presented positive values, showing that all variables are positively correlated with the factor. In addition to the positive sign of the factor loadings, the values ranged between 0.555 and 0.911, indicating magnitudes that vary from moderate to high. In general, factor loadings above 0.50 are considered adequate to indicate that the item represents the latent construct, while values above 0.70 reflect a strong association with the factor. Thus, most items showed high representativeness of the proposed unidimensional factor, with only three items being close to the minimum acceptable limit (loadings between 0.55 and 0.57), but still within standards considered satisfactory in confirmatory models for psychometric instruments.

**Table 4:** Calculation of factor loadings. São Carlos, São Paulo, Brazil, 2024

	Estimate	Standard error	*p* value
Item 1	0.891	0.020	0.000
Item 2	0.851	0.027	0.000
Item 3	0.869	0.025	0.000
Item 4	0.810	0.033	0.000
Item 5	0.868	0.025	0.000
Item 6	0.688	0.049	0.000
Item 7	0.736	0.045	0.000
Item 8	0.911	0.020	0.000
Item 9	0.555	0.061	0.000
Item 10	0.765	0.037	0.000
Item 11	0.555	0.063	0.000
Item 12	0.565	0.059	0.000

The mean score for the Brazilian version of Teacher’s Self-Efficacy in Asthma Management was 29.86 (SD = 11.89; median = 28; minimum = 12; maximum = 60). The floor/ceiling effects were 5.59% and 0.70%, respectively, both within the acceptable limit of 15%. Internal consistency, assessed using Cronbach’s alpha coefficient, was 0.922, indicating high reliability of the instrument.

Finally, Table 5 presents a comparison between the mean self-efficacy score for asthma management and the variables age group and years of training. It can be observed that, on average, teachers with less than five years of training had scores 24% lower compared to those with more than five years of training.

**Table 5:** Comparison between the mean self-efficacy score for asthma management and the variables age group and years of training. São Carlos, São Paulo, Brazil, 2024

Variables	Mean (SD)	Median (Min.-Max.)	Estimated effect (95%CI)	*p* value
*Age group*				
<40 anos (n = 59)	27.68 (11.31)	25 (12-60)	0.88 (0.77;1.01)	0.06
>40 anos (n = 84)	31.39 (12.11)	30 (12-57)	ref.	
*Years since graduation*				
<5 anos (n = 14)	23.21 (9.8)	21.5 (12-47)	0.76 (0.61;0.95)	0.02
>5 anos (n = 129)	30.58 (11.9)	30 (12-60)	ref.	

*Legend: SD - standard deviation; Min.-Max. - Minimum-Maximum; 95%CI - 95% Confidence Interval.*

**Figure 1:**
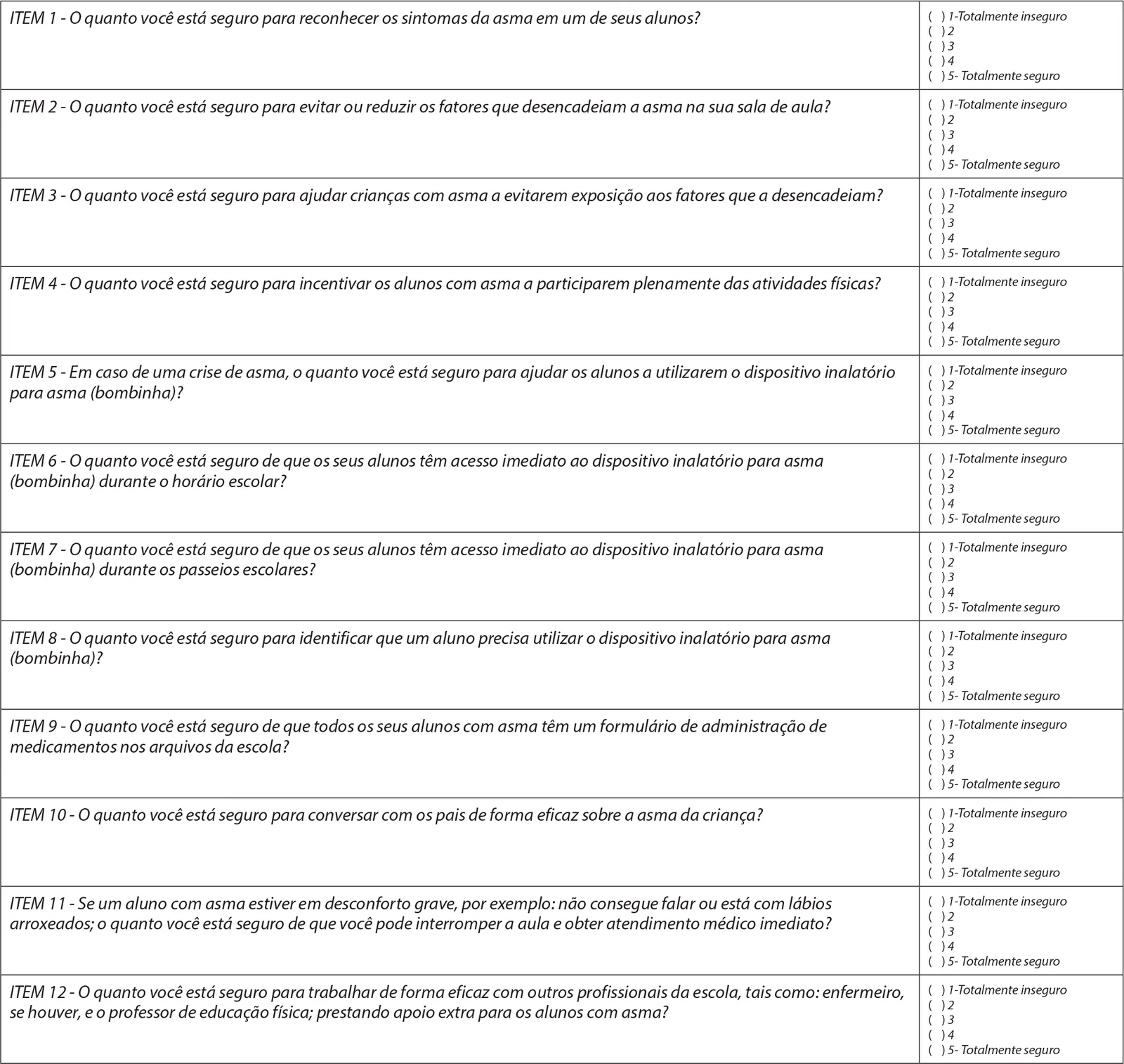
Brazilian version of Teacher’s Self-Efficacy in Asthma Management. São Carlos, São Paulo, Brazil, 2024

The Brazilian version of Teacher’s Self-Efficacy in Asthma Management is available in Figure 1.

## DISCUSSION

The cross-cultural adaptation stages were carried out in accordance with recommendations widely used in methodological and psychometric studies developed in nursing^(22,23)^. After the initial stages of translation, translation synthesis, and back-translation, the scale was submitted to a committee of experts, where the “semantic equivalence” criterion obtained a CVI of less than 0.80 in four items. According to a psychometric study conducted in Turkey, which aimed to translate and adapt the same scale with 216 teachers, the CVI ranged from 0.87 to 0.99 in all items^(24)^. However, this difference does not invalidate the findings of the present investigation, since all the recommendations proposed by experts were met until consensus was reached. The recommendations aimed primarily to facilitate the understanding of technical health terms among laypeople. These adjustments had a positive impact on the pre-final version test, as the majority (76.7%) considered the scale easy to understand.

The factor structure was acceptable and confirmed the unidimensionality of the scale. The calculation of several indices to assess the quality of the factor solution aligns with contemporary validity evidence recommendations, which emphasize the importance of models tested using multiple techniques to improve the accuracy and quality of instruments^(25,26)^.

In CFA, the CFI and TLI values were above 0.90, indicating a good model fit. The RMSEA value was above the acceptable level, being 0.176 in the initial model and 0.090 in the re-specified model, demonstrating a poor fit. The article presenting the original version of the scale did not perform CFA, making it impossible to compare the fit indices. In contrast, the Turkish research that also validated this scale obtained an RMSEA of less than 0.06, indicating a good fit^(24)^. One hypothesis to explain this discrepancy could be the number of participants, since the present study included 143 teachers, and the Turkish study had 206, because, in small samples, RMSEA tends to erroneously indicate a pessimistic adjustment model^(27)^.

Despite the limitations reflected by RMSEA, the WRMR index was 0.652 in the re-specified model, indicating an acceptable fit. The literature highlights that WRMR indices below 1 are considered acceptable, and when assessed together with other psychometric metrics, such as CFI and TLI, support the model’s suitability to the new cultural reality, serving as important signals of the quality of the fit^(28)^.

Similarly, the analysis of the residual polychoric correlation matrix revealed minimal discrepancies between the observed correlations and those estimated by the theoretical model, with values ranging from -0.1 to 0.1. These findings suggest that the model successfully represented the interactions among scale elements, reducing any deviations that could affect the interpretation of results^(29)^. Small residual discrepancies indicate a high adequacy of the theoretical model to empirical data, reinforcing the structural validity of the adapted scale^(30)^.

Floor/ceiling effects are frequently used as quality indicators in methodological studies. The occurrence of high floor/ceiling effects may indicate flaws in the scale design. On the other hand, when these effects are low, as observed in the present study, the scale is considered well-adjusted, being able to accurately capture differences in perceptions, in this case, in the self-efficacy levels of participants^(31)^.

The floor/ceiling effects were within acceptable limits in the literature, indicating that the scale was able to distinguish the different perceptions of self-efficacy among participants without restricting variability in responses. A systematic review that assessed the usefulness of pediatric asthma control questionnaires highlighted the challenge of assessing the methodological quality of these instruments. Among the criteria adopted was the analysis of floor/ceiling effects, and in this study, three questionnaires were classified as having low quality in this aspect^(32)^.

In relation to the internal consistency of the adapted version, Cronbach’s alpha coefficient was 0.922. This result is considered excellent, as it exceeds the minimum acceptable limit in the literature of 0.70, demonstrating that the questions are highly correlated with each other and present stable and reliable measurement of the same construct^(19)^. Furthermore, it suggests that participants understood the items well. In the original version of the scale, as well as in the Turkish version, Cronbach’s alpha was 0.88 and 0.70, respectively^(7,24)^.

Another relevant aspect concerns participants with less than five years of training, who, on average, presented a self-efficacy score 24% lower than that observed among professionals with more experience. Conversely, a study that investigated the level of knowledge of basic education teachers about asthma found that teachers with less experience obtained higher knowledge scores^(33)^. Although they are distinct constructs, the comparison is justified, since knowledge can be considered a factor potentially associated with self-confidence and the perception of security in managing health situations in a school setting.

On the other hand, the age variable did not show a statistically significant difference. In contrast, a study on teachers’ knowledge and attitudes towards childhood diabetes, another chronic condition, revealed that teachers over 45 years of age demonstrated a greater understanding of how to manage the disease, a result attributed to the experience accumulated over years of professional practice^(34)^.

### Study limitations

Although the results of this study are supported by the literature, it is worth pointing out its limitations. The first refers to the number of participants, which may not be representative of all Brazilian teachers, especially considering regional, cultural, and educational infrastructure variations. Another limitation was the lack of analysis of concurrent criterion validity, since no instruments were found that assessed the same phenomenon. These limitations do not compromise the relevance of the study, but offer opportunities for improvements in future studies or additional validity stages.

### Contributions to nursing

This scale contributes to nursing, especially because it can be used as a situational analysis tool capable of supporting the development of training and updating programs aimed at teachers, contributing to strengthening the integration between health and education.

## CONCLUSION

The Brazilian Portuguese version of Teachers’ Self-Efficacy in Asthma Management presented a unidimensional characteristic, satisfactory factor loadings, and good levels of reliability, which point to an instrument with evidence of a consistent and reliable internal structure for measuring the desired construct.

## Data Availability

Available research data is in the body of the article.
