# Supplementary material for: Cross-cultural adaptation and psychometric analysis of Teachers’ Self-Efficacy in Asthma Management
Source: Rev Bras Enferm. 2026 Sep 21;79:e20250438. doi: 10.1590/0034-7167-2025-0438 (PMC13592786; doi:10.1590/0034-7167-2025-0438)
Supplement: Translation

# Translation: Adaptação transcultural e análise psicométrica da escala *Teachers’ Self-Efficacy in Asthma Management*

**DOI:** 10.1590/0034-7167-2025-0438pt

**Authors:** Francisco Figueiredo, Bruna Josiane de Lima, Ingrid Daiane Silva Aiello, Aline Helena Appoloni Eduardo, Edmara Bazoni Soares Maia, Aline Cristiane Cavicchioli Okido

**Affiliations:** I Universidade Federal de São Carlos. São Carlos, São Paulo, Brasil; II Universidade Federal de São Paulo. São Paulo, São Paulo, Brasil; III Universidade de São Paulo. Ribeirão Preto, São Paulo, Brasil

**Keywords:** Estudos de Validação, Autoeficácia, Asma, Criança, Professores Escolares.

## Abstract

**Objetivo:**

realizar a adaptação transcultural e a análise psicométrica da escala *Teachers’ Self-Efficacy in Asthma Management* para o contexto brasileiro.

**Métodos:**

estudo psicométrico, abrangendo as etapas de tradução, síntese das traduções, retrotradução, submissão a um comitê de especialistas, teste da versão pré-final e teste da versão adaptada. Para análise da validade de construto e confiabilidade, realizaram-se análise fatorial confirmatória e cálculo do coeficiente de alfa de Cronbach.

**Resultados:**

o Índice de Validação de Conteúdo da versão traduzida foi superior a 0,80, após modificações propostas pelos especialistas. O teste da versão pré-final indicou boa compreensão e facilidade no processo de resposta. A versão adaptada foi testada em 143 professores. A análise fatorial confirmatória demonstrou bom ajuste no modelo, com cargas fatoriais entre 0,555 e 0,911. O coeficiente alfa de Cronbach foi de 0,922.

**Conclusão:**

a versão brasileira da escala apresentou medidas psicométricas confiáveis e válidas, confirmando sua unidimensionalidade.

## INTRODUÇÃO

A asma está entre as doenças crônicas mais prevalentes na população infantil, acometendo mais de 300 milhões de pessoas em todo o mundo^(1)^. Nos Estados Unidos, estima-se que, atualmente, 4,6 milhões de crianças e adolescentes possuem esse diagnóstico^(2)^. No Brasil, estudo realizado com 16.237 crianças atendidas em um ambulatório de um hospital pediátrico de referência identificou a asma como o diagnóstico mais frequente entre os casos analisados^(3)^.

O principal objetivo do manejo da asma é reduzir o risco de exacerbações e melhorar a qualidade de vida dos indivíduos^(4)^. Entre as medidas não farmacológicas que contribuem para o controle dos sintomas e a melhora da qualidade de vida em crianças asmáticas, destacam-se evitar a exposição ao tabaco, reduzir o contato com ambientes poluídos, promover a perda de peso em crianças com sobrepeso e incentivar a prática de exercícios respiratórios^(5)^. O manejo farmacológico, por sua vez, deve ser orientado conforme a gravidade clínica e o nível de controle da doença. Apesar da disponibilidade de tratamentos eficazes, a asma não controlada permanece altamente prevalente, exigindo, frequentemente, atendimento hospitalar. Nesse sentido, segundo dados do Departamento de Informação e Informática do Sistema Único de Saúde, conhecido como DATASUS, em 2019, o Brasil registrou 53.352 internações de crianças entre 0 e 14 anos por asma, sendo 8.074 apenas no estado de São Paulo^(6)^.

Considerando que a asma é prevalente na faixa etária de 5 a 14 anos, que essas crianças permanecem, em média, um terço do dia no ambiente escolar e que a maioria das escolas não conta com a presença de profissionais de enfermagem, a literatura destaca a importância do papel dos professores na promoção do bem-estar dessas crianças em sala de aula. Além disso, ressalta-se a necessidade de que reconheçam sinais de asma não controlada, uma vez que a exposição a gatilhos ao longo do período escolar pode desencadear crises graves e, potencialmente, fatais^(4)^. No entanto, a ausência de treinamentos específicos e a falta de conhecimento adequado para lidar com emergências relacionadas à asma geram desconforto e insegurança entre os professores^(7)^.

O manejo de uma criança com sintomas de asma pode representar uma experiência extremamente desafiadora e angustiante, especialmente na presença de lacunas de conhecimento. Essa condição impacta negativamente a percepção de autoeficácia dos professores, comprometendo sua capacidade de agir de forma rápida e assertiva diante da situação^(8)^. O conceito de autoeficácia, introduzido por Bandura em 1977, refere-se à confiança do indivíduo em sua própria capacidade de executar determinada ação com sucesso e alcançar os resultados desejados^(9)^.

Nos Estados Unidos, cerca de 45% das escolas contam com a presença de um enfermeiro em tempo integral^(10)^. No Brasil, a presença de um profissional de enfermagem exclusivo é observada apenas em algumas instituições de ensino privadas. É nesse contexto que se insere o Programa Saúde na Escola, uma política pública brasileira criada para atender às demandas locais por meio da articulação intersetorial entre o setor educacional e as unidades da Estratégia Saúde da Família. No entanto, o potencial resolutivo do programa tem apresentado limitações significativas que dificultam sua expansão e efetividade. Entre os principais desafios, destacam-se lacunas na formação profissional, fragilidades no monitoramento das informações, ausência de planejamento articulado entre as equipes, falhas na comunicação, carência de infraestrutura e insuficiência de investimentos públicos^(11)^.

Seguindo essa perspectiva, pesquisadoras norte-americanas desenvolveram a escala intitulada *Teachers’ Self-Efficacy in Asthma Management*. O instrumento é composto por 12 itens, que avaliam a autoeficácia dos professores em diferentes dimensões do manejo da asma, incluindo o reconhecimento de sintomas, prevenção de gatilhos, administração de medicamentos, comunicação com os pais, incentivo à participação em atividades físicas e manejo de situações de emergência. A escala adota o formato Likert de cinco pontos, com respostas que variam de 1 (totalmente inseguro) a 5 (totalmente seguro). A pontuação final é obtida pela soma dos escores atribuídos a cada item, podendo variar entre 12 e 60 pontos. Quanto maior a pontuação obtida, maior o nível de autoeficácia percebido pelo professor^(7)^.

Diante do exposto, e considerando a escassez de estudos que abordem a autoeficácia de professores no manejo da asma, justifica-se a originalidade do presente estudo ao propor a adaptação transcultural e a análise psicométrica da escala *Teachers’ Self-Efficacy in Asthma Management*, uma vez que não há, até o momento, instrumento equivalente validado para o contexto brasileiro. Ademais, ressalta-se a relevância social da proposta, ao disponibilizar uma ferramenta com potencial para subsidiar o planejamento e a implementação de estratégias educativas sistematizadas e eficazes voltadas aos professores, contribuindo para o cuidado e a segurança de crianças com asma no ambiente escolar.

## OBJETIVO

Realizar a adaptação transcultural e análise psicométrica da escala *Teachers’ Self-Efficacy in Asthma Management* para o contexto brasileiro.

## MÉTODOS

### Aspectos éticos

O projeto de pesquisa foi submetido à apreciação e aprovado por Comitê de Ética em Pesquisa, em conformidade com os princípios éticos estabelecidos pela Resolução nº 510/2016 do Conselho Nacional de Saúde. Ressalta-se, ainda, que houve contato prévio com a pesquisadora responsável pela elaboração do instrumento original, a qual autorizou formalmente a realização deste estudo no Brasil.

### Desenho, período e local do estudo

Trata-se de estudo psicométrico que percorreu as etapas de tradução inicial, síntese das traduções, retrotradução, submissão a um comitê de especialistas, teste da versão pré-final e teste da versão adaptada^(12)^. Todo o percurso metodológico foi realizado entre março de 2022 e agosto de 2024. As quatro primeiras etapas foram conduzidas por meio de comunicação eletrônica entre pesquisadores, tradutores e membros do comitê de especialistas. O teste da versão pré-final foi realizado em escolas públicas e privadas de educação infantil, ensino fundamental e ensino médio de um município do interior do estado de São Paulo. Já a aplicação da versão adaptada foi conduzida de forma remota, sem delimitação geográfica para a coleta de dados, o que possibilitou a participação de professores de diferentes localidades do país.

### População e amostra: critérios de inclusão e exclusão

As etapas de tradução, síntese e retrotradução contaram com a participação de dois tradutores independentes, brasileiros, fluentes em ambos os idiomas. Quanto ao comitê de especialistas, os critérios de inclusão abrangeram profissionais com *expertise* na área de asma no âmbito pediátrico, com experiência acadêmica e/ou clínico-assistencial, bem como pesquisadores com vivência em estudos de adaptação cultural. Os critérios de elegibilidade para as quinta e sexta etapas incluíram professores maiores de 18 anos, com pelo menos um ano de atuação em escolas públicas ou privadas. Não foram estabelecidos critérios de exclusão.

### Coleta e organização dos dados

#### Tradução inicial, síntese das traduções e retrotradução

Inicialmente, a escala foi traduzida para a língua portuguesa por dois tradutores independentes. Em seguida, o grupo de pesquisa se reuniu para analisar as duas versões produzidas e, de forma consensual, elaborar uma síntese das traduções. A síntese resultante foi então retrotraduzida para o inglês, a fim de ser comparada com a versão original do instrumento.

#### Submissão a um comitê de especialistas

Os especialistas foram recrutados por meio de consulta à plataforma *Lattes* e pela estratégia *snowball sampling* ^(13)^. Os profissionais elegíveis receberam convite para participar da pesquisa via e-mail, contendo um *link* que os direcionava à plataforma *Google Forms^®^*. O Termo de Consentimento Livre e Esclarecido (TCLE) foi disponibilizado na página inicial do formulário, e somente após manifestação de concordância, os especialistas tiveram acesso à versão consensual da escala para avaliação.

O comitê de especialistas avaliou a versão traduzida considerando as equivalências conceitual, operacional e semântica dos itens, utilizando uma escala Likert com as seguintes opções de resposta: -1 (não concordo), 0 (indeciso) e 1 (concordo). Os participantes foram orientados a comentar e sugerir melhorias sempre que atribuíssem as respostas -1 ou 0. Ademais, responderam questões para caracterização do perfil, como área de formação, idade, tempo de formação, titulação e área de atuação profissional. O tempo estimado para análise e preenchimento do formulário foi de aproximadamente 30 minutos.

#### Teste da versão pré-final

Esta etapa teve como objetivo verificar a aceitabilidade e a compreensão do instrumento pelo público-alvo, ou seja, os professores. A compreensão adequada dos itens é fundamental para evitar confusões quanto ao significado geral da escala e erros de interpretação de termos ou conceitos específicos.

Para tanto, foi estabelecido contato com diretores e/ou coordenadores de escolas públicas e privadas de educação infantil, ensino fundamental e médio do município onde a pesquisa foi realizada, com o intuito de apresentar o projeto e solicitar autorização para seu desenvolvimento. Após a obtenção da autorização, os pesquisadores convidaram presencialmente os potenciais participantes. Aqueles que concordaram em participar responderam a um questionário de caracterização sociodemográfica (sexo, idade, tempo de formação, presença de diagnóstico de asma ou familiar asmático), além de responder à versão da escala validada pelo comitê de especialistas.

Em seguida, os participantes realizaram uma análise geral da escala, indicando se os itens estavam claros e compreensíveis, bem como se encontraram alguma dificuldade na resposta. Houve espaço para sugestões e comentários. Por fim, os professores foram convidados a analisar especificamente quatro itens da escala. Para isso, os 12 itens foram divididos aleatoriamente em três subconjuntos de quatro itens cada, e os participantes foram distribuídos entre esses grupos. Essa análise foi conduzida por meio de entrevistas gravadas, nas quais os professores foram solicitados a reformular com suas próprias palavras cada item. Essa abordagem permitiu aos pesquisadores avaliar o grau de compreensão dos participantes e identificar termos ou expressões que pudessem contribuir para maior clareza do instrumento. Tal estratégia se baseou nas recomendações e instrumentos do DISABKIDS^®(14)^.

#### Teste da versão adaptada

Inicialmente, foi elaborado um cartaz contendo o objetivo do estudo e o convite direcionado aos professores. A divulgação do cartaz foi realizada por meio de envio por e-mail, compartilhamento em redes sociais (como grupos de *WhatsApp* ^®^ e *Facebook* ^®^) e fixação em murais de escolas do município de origem do pesquisador. Os professores interessados em participar acessaram o formulário de pesquisa por meio de um *QR Code* ou *link* disponibilizado no próprio cartaz.

Ao ingressar no formulário, os participantes tinham, primeiramente, acesso ao TCLE. Somente após a manifestação de concordância com os termos é que se iniciava o preenchimento da escala, juntamente com o questionário de caracterização, que incluía informações como idade, tempo de formação, área de atuação e tipo de instituição de ensino. Todos os professores que acessaram o TCLE concordaram em participar da pesquisa, não havendo recusas registradas. O tempo médio estimado para o preenchimento do instrumento foi de aproximadamente cinco minutos.

### Análise dos dados

Para a análise dos dados referentes à quarta etapa (submissão a um comitê de especialistas), foram consideradas as sugestões dos avaliadores, calculando-se o Índice de Validação de Conteúdo (IVC) por item em cada um dos aspectos analisados, como equivalência conceitual, equivalência dos itens, equivalência operacional e equivalência semântica. Para fins de validação do conteúdo, foi considerado adequado um IVC igual ou superior a 0,80^(15)^.

Para examinar as propriedades psicométricas da versão adaptada, foi utilizado exclusivamente um modelo de análise fatorial confirmatória (AFC), uma vez que o objetivo era testar a estrutura unidimensional previamente definida no instrumento original, não havendo, portanto, a realização de análise fatorial exploratória. A extração dos fatores seguiu o estimador *Weighted Least Squares Mean and Variance Adjusted*, apropriado para itens ordinais e baseado em matriz de correlação policórica. Com relação aos modelos testados, foi estimado apenas um modelo confirmatório, correspondente à estrutura unifatorial originalmente proposta para o instrumento, de forma a avaliar a adequação do modelo teórico à amostra estudada.

Para avaliar o ajuste dos modelos aos dados, foram utilizados os seguintes índices: qui-quadrado (χ^2^); *Comparative Fit Index* (CFI); *Tucker-Lewis Index* (TLI); *Root Mean Square Error of Approximation* (RMSEA); e *Weighted Root Mean Square Residual* (WRMR). Foram considerados indicativos de bom ajuste os valores de CFI e TLI ≥ 0,90^(16)^, RMSEA ≤ 0,06^(17)^ e WRMR < 1,0^(18)^. A matriz de correlação policórica residual foi analisada com o intuito de identificar possíveis subestimações ou superestimações das relações entre variáveis, sendo considerados aceitáveis os resíduos entre -0,1 e 0,1.

Após a AFC, os dados foram descritos por meio de frequências absolutas e percentuais, para variáveis qualitativas, bem como por meio de medidas de tendência central e dispersão, como média, desvio padrão (DP), mínimo, mediana e máximo, para variáveis quantitativas. A confiabilidade foi avaliada por meio da consistência interna, utilizando o coeficiente alfa de Cronbach, sendo considerado satisfatório um valor superior a 0,70^(19)^.

Os efeitos piso e teto (*floor*/*ceiling*) foram analisados com base na porcentagem de participantes que atingiram, respectivamente, a pontuação mínima ou máxima possível. Considera-se que há efeito relevante quando mais de 15% dos participantes alcançam uma das pontuações extremas^(20)^. Para comparar a pontuação total de autoeficácia segundo as variáveis de interesse (faixa etária e tempo de formação), foi empregado um modelo de regressão com distribuição binomial negativa e função de ligação logarítmica^(21)^.

Todos os gráficos foram elaborados com o auxílio do *software* RStudio, versão 2023.12.0.369, enquanto as análises estatísticas foram conduzidas no *software* SAS 9.4, com exceção da AFC, realizada no *software* MPLUS 6.12. Para todas as análises, adotou-se um nível de significância de 5%. As análises estatísticas contaram com o apoio de um profissional da área.

## RESULTADOS

O comitê de especialistas foi composto por 24 profissionais de medicina (pediatria), enfermagem e fisioterapia. A média de idade dos participantes foi de 35,5 anos (DP = 7,3), com tempo médio de formação de 10,9 anos (DP = 3,5). Em relação à titulação acadêmica, 33,3% possuíam doutorado, 23,8%, mestrado, e 42,9% eram especialistas. No que se refere à atuação profissional, 28,6% exerciam atividades docentes, e 71,4% atuavam na assistência à saúde.

O IVC para o critério “equivalência semântica” foi inferior ao limite estabelecido em dez itens. Nos demais critérios avaliados, todos os itens atingiram índices superiores a 0,80 já na primeira rodada. Entre as principais sugestões dos especialistas para aprimorar a clareza dos itens, destacam-se a substituição de “gatilho” por “fatores que desencadeiam a asma”, “inalador” por “dispositivo inalatório para asma (bombinha)”, “ataque de asma” por “crise de asma” e “lábios azuis” por “lábios arroxeados”. Com base nessas sugestões, uma nova versão da escala foi elaborada, incorporando as modificações propostas, e submetida a uma segunda rodada de avaliação, na qual foi alcançado o consenso entre os especialistas.

No teste da versão pré-final, participaram 30 professores, majoritariamente do sexo feminino, com média de idade de 37,9 anos (DP = 9,4) e tempo médio de formação de 11,2 anos (DP = 4,1). Um professor afirmou ter o diagnóstico de asma, e 27,3% possuíam familiar asmático. A Tabela 1 apresenta a avaliação geral do instrumento, evidenciando que a maioria considerou a escala “muito boa” e “fácil de entender”. Sobre as categorias de resposta, 86,7% indicaram não ter encontrado dificuldade, e todos consideraram as questões como “muito importantes”.

**Tabela 1:** Distribuição dos professores (N = 30) segundo categoria de resposta do formulário de impressão geral. São Carlos, São Paulo, Brasil, 2024

Itens do formulário	n (%)
**O que você acha do nosso instrumento em geral?**	
Muito bom	17 (56,7)
Bom	13 (43,3)
Regular/mais ou menos	0 (0)
**O que você achou das questões?**	
Todas foram fáceis	23 (76,7)
Algumas foram difíceis	07 (23,3)
Todas foram difíceis	0 (0)
**Sobre as categorias de resposta, você teve alguma dificuldade em usá-las?**	
Sem dificuldades	26(86,7)
Algumas dificuldades	04 (13,3)
Muitas dificuldades	0 (0)
**As questões são importantes para a condição de saúde/doença dos seus alunos?**	
Muito importante	30 (100)
Às vezes importante	0 (0)
Nada importante	0 (0)
**Você gostaria de mudar algo no instrumento?**	
Sim	2 (6,7)
Não	28 (93,3)
**Você gostaria de acrescentar algo no instrumento?**	
Sim	03 (10)
Não	27 (90)
**Houve alguma questão que você não quis responder? Se sim, por quê?**	
Sim	01 (3,3)
Não	29 (96,7)

Na análise específica, verificou-se que, de modo geral, os participantes foram capazes de explicar adequadamente os itens da escala, o que indica um nível satisfatório de compreensão. A partir da análise do material empírico, os pesquisadores identificaram a recorrência de determinadas expressões utilizadas pelos participantes, caracterizadas por uma linguagem mais clara e acessível. Com base nisso, foram realizadas as seguintes substituições na versão final do instrumento: “o quão seguro” foi substituído por “o quanto você está seguro” (em todos os itens); “avaliar se” por “identificar que” (no item 8); e “comunicar com os pais” por “conversar com os pais” (no item 10).

A versão adaptada foi aplicada a 143 professores. Desses, 84 (58,7%) tinham mais de 40 anos de idade, e o tempo médio de formação foi de 17,43 anos (DP = 10,25), variando de 1 a 42 anos. Em relação à área de atuação, 26 docentes (18,2%) atuavam na educação infantil, 28 (19,6%), no ensino fundamental I, nove (6,3%), no ensino fundamental II, 16 (11,2%), no ensino médio, e 64 (44,8%), em mais de uma etapa de ensino. Quanto ao tipo de instituição, 124 participantes (86,7%) atuavam em escolas públicas.

A Tabela 2 apresenta os valores obtidos para os índices de ajuste considerando um modelo unifatorial: χ^2^; CFI; TLI; RMSEA; e WRMR. Observa-se que os valores de CFI e TLI foram superiores a 0,90, indicando um bom ajuste do modelo. Em contrapartida, o valor de RMSEA esteve acima do limite aceitável, sugerindo um ajuste insatisfatório. Por fim, o valor de WRMR no modelo reespecificado foi de 0,652, o que indica um ajuste aceitável.

**Tabela 2:** Descrição dos índices estatísticos iniciais. São Carlos, São Paulo, Brasil, 2024

Índices	Modelo inicial	Modelo re-especificado
RMSEA	0,176 (IC90%: 0,156-0,196)	0,090 (IC90%: 0,066-0,114)
CFI	0,943	0,987
TLI	0,931	0,982
χ^2^	291,934 (54)	103,236 (48)
WRMR	1,361	0,652

*Legenda: RMSEA - Root Mean Square Error of Approximation; CFI - Comparative Fit Index; TLI - Tucker-Lewis Index; χ^2^ - qui-quadrado; WRMR - Weighted Root Mean Square Residual; IC90% - Intervalo de Confiança de 90%.*

A Tabela 3 apresenta, a seguir, a matriz de correlação policórica residual, obtida a partir das discrepâncias entre as correlações observadas e as estimadas. Verifica-se que os resíduos permaneceram no intervalo de -0,1 a 0,1, não indicando evidências de subestimação ou superestimação nas relações entre as variáveis.

**Tabela 3:** Matriz de correlação residual. São Carlos, São Paulo, Brasil, 2024

	Item 1	Item 2	Item 3	Item 4	Item 5	Item 6	Item 7	Item 8	Item 9	Item 10	Item 11
**Item 2**	0,031										
**Item 3**	0,030	0,000									
**Item 4**	-0,009	0,018	0,041								
**Item 5**	-0,018	-0,037	-0,023	-0,017							
**Item 6**	-0,058	0,065	-0,060	-0,015	0,000						
**Item 7**	-0,087	0,021	-0,074	0,007	0,079	0,000					
**Item 8**	-0,007	-0,022	-0,001	-0,093	0,038	0,007	-0,050				
**Item 9**	-0,062	0,015	-0,095	-0,030	-0,064	0,124	0,126	-0,008			
**Item 10**	0,032	-0,070	0,006	-0,009	-0,027	-0,060	-0,052	0,040	0,000		
**Item 11**	-0,006	-0,019	0,063	0,052	-0,020	-0,062	-0,107	0,030	-0,144	0,048	
**Item 12**	-0,042	-0,021	-0,021	0,089	-0,083	0,062	0,077	-0,062	0,049	0	0

A seguir, foram calculadas as cargas fatoriais (Tabela 4), que indicam a correlação de cada variável com o fator. Observa-se que todas as cargas fatoriais apresentaram valores positivos, evidenciando que todas as variáveis estão positivamente correlacionadas com o fator. Além do sinal positivo das cargas fatoriais, observa-se que os valores ficaram entre 0,555 e 0,911, indicando magnitudes que variam de moderadas a altas. De modo geral, cargas fatoriais acima de 0,50 são consideradas adequadas para indicar que o item representa o construto latente, enquanto valores superiores a 0,70 refletem forte associação com o fator. Assim, a maior parte dos itens apresentou elevada representatividade do fator unidimensional proposto, com apenas três itens, situando-se próximos ao limite mínimo aceitável (cargas entre 0,55 e 0,57), porém ainda dentro de padrões considerados satisfatórios em modelos confirmatórios para instrumentos psicométricos.

**Tabela 4:** Cálculo das cargas fatoriais. São Carlos, São Paulo, Brasil, 2024

	Estimativa	Erro padrão	Valor de *p*
Item 1	0,891	0,020	0,000
Item 2	0,851	0,027	0,000
Item 3	0,869	0,025	0,000
Item 4	0,810	0,033	0,000
Item 5	0,868	0,025	0,000
Item 6	0,688	0,049	0,000
Item 7	0,736	0,045	0,000
Item 8	0,911	0,020	0,000
Item 9	0,555	0,061	0,000
Item 10	0,765	0,037	0,000
Item 11	0,555	0,063	0,000
Item 12	0,565	0,059	0,000

A pontuação média da versão brasileira da *Teacher’s Self-Efficacy in Asthma Management* foi de 29,86 (DP = 11,89; mediana = 28; mínimo = 12; máximo = 60). Os efeitos piso e teto foram, respectivamente, de 5,59% e 0,70%, ambos dentro do limite aceitável de 15%. A consistência interna, avaliada por meio do coeficiente alfa de Cronbach, foi de 0,922, indicando elevada confiabilidade do instrumento.

Por fim, a Tabela 5 apresenta a comparação entre a pontuação média de autoeficácia para o manejo da asma e as variáveis faixa etária e tempo de formação. Observa-se que, em média, professores com menos de cinco anos de formação apresentaram pontuação 24% inferior em comparação àqueles com mais de cinco anos de formação.

**Tabela 5:** Comparação entre a pontuação média de autoeficácia para manejo da asma e as variáveis faixa etária e tempo de formação. São Carlos, São Paulo, Brasil, 2024

Variáveis	Média (DP)	Mediana (Mín.-Máx.)	Efeito estimado (IC95%)	Valor de *p*
*Faixa etária*				
<40 anos (n = 59)	27,68 (11,31)	25 (12-60)	0,88 (0,77;1,01)	0,06
>40 anos (n = 84)	31,39 (12,11)	30 (12-57)	ref.	
*Tempo de formação*				
<5 anos (n = 14)	23,21 (9,8)	21,5 (12-47)	0,76 (0,61;0,95)	0,02
>5 anos (n = 129)	30,58 (11,9)	30 (12-60)	ref.	

*Legenda: DP - desvio padrão; Mín.-Máx. - Mínimo-Máximo; IC95% - Intervalo de Confiança de 95%.*

A versão brasileira da *Teacher’s Self-Efficacy in Asthma Management* está disponível na Figura 1.

**Figura 1:** Versão brasileira da Teacher’s Self-Efficacy in Asthma Management. São Carlos, São Paulo, Brasil, 2024

## DISCUSSÃO

As etapas de adaptação transcultural foram realizadas de acordo com recomendações amplamente utilizadas em estudos metodológicos e psicométricos desenvolvidos na área da enfermagem^(22,23)^. Após as etapas iniciais de tradução, síntese das traduções e retrotradução, a escala foi submetida a um comitê de especialistas, em que o critério “equivalência semântica” obteve IVC inferior a 0,80 em quatro itens. De acordo com estudo psicométrico realizado na Turquia, cujo objetivo foi traduzir e adaptar a mesma escala junto a 216 professores, o IVC variou de 0,87 a 0,99 em todos os itens^(24)^. Todavia, essa diferença não invalida os achados da presente investigação, uma vez que todas as recomendações propostas pelos especialistas foram atendidas até o consenso. As recomendações tinham como principal intenção facilitar o entendimento de termos técnicos da área da saúde entre leigos. Tais ajustes repercutiram positivamente no teste da versão pré-final à medida que a maioria (76,7%) considerou a escala fácil de entender.

A estrutura fatorial foi aceitável e confirmou a unidimensionalidade da escala. O cálculo de vários índices para avaliar a qualidade da solução fatorial se alinha às recomendações contemporâneas de evidências de validade, que pontuam a importância de modelos testados em múltiplas técnicas para melhorar a precisão e a qualidade dos instrumentos^(25,26)^.

Na AFC, os valores de CFI e TLI ficaram acima de 0,90, indicando um bom ajuste do modelo. O valor do RMSEA foi acima do aceitável, sendo 0,176, no modelo inicial, e 0,090, no modelo re-especificado, demonstrando um ajuste pobre. O artigo que apresenta a versão original da escala não realizou o AFC, impossibilitando a comparação entre os índices de ajuste. Em contrapartida, a pesquisa turca que também validou esta escala obteve RMSEA menor de 0,06, indicando um bom ajuste^(24)^. Uma hipótese para justificar essa discrepância pode ser o número de participantes, uma vez que o presente estudo contou com 143 professores, e no estudo turco, foram 206, pois, em amostras pequenas, o RMSEA tende a indicar erroneamente um modelo de ajuste pessimista^(27)^.

Apesar das limitações refletidas pelo RMSEA, o índice WRMR foi de 0,652 no modelo re-especificado, indicando um ajuste aceitável. A literatura ressalta que índices de WRMR abaixo de 1 são vistos como aceitáveis, e quando avaliados juntamente com outras métricas psicométricas, como CFI e TLI, apoiam a adequação do modelo à nova realidade cultural, servindo como sinais importantes da qualidade do ajuste^(28)^.

Na mesma direção, a análise da matriz de correlação policórica residual revelou discrepâncias mínimas entre as correlações observadas e aquelas estimadas pelo modelo teórico, com valores variando entre -0,1 e 0,1. Esses achados sugerem que o modelo conseguiu representar bem as interações entre os elementos da escala, reduzindo eventuais desvios que poderiam afetar a interpretação dos resultados^(29)^. Discrepâncias residuais pequenas indicam uma elevada adequação do modelo teórico aos dados empíricos, reforçando a validade estrutural da escala adaptada^(30)^.

Os efeitos de piso e teto são frequentemente utilizados como indicadores da qualidade em estudos metodológicos. A ocorrência de altos efeitos de piso e teto pode indicar falhas no planejamento da escala. Por outro lado, quando esses efeitos são baixos, como observado no presente estudo, a escala é considerada bem ajustada, sendo capaz de captar com precisão as diferenças nas percepções, neste caso, nos níveis de autoeficácia dos participantes^(31)^.

Os efeitos de piso e teto ficaram dentro do limite aceitável na literatura, indicando que a escala conseguiu distinguir as diferentes percepções de autoeficácia dos participantes sem restringir a variabilidade nas respostas. Revisão sistemática que avaliou a utilidade de questionários de controle de asma pediátrica destacou o desafio de avaliar a qualidade metodológica desses instrumentos. Entre os critérios adotados, incluiu-se a análise dos efeitos de piso e teto, sendo que, nesse estudo, três questionários foram classificados como de baixa qualidade nesse aspecto^(32)^.

Quanto à consistência interna da versão adaptada, o coeficiente alfa de Cronbach foi de 0,922. Esse resultado é considerado excelente, pois supera o limite mínimo aceitável na literatura de 0,70, demonstrando que as questões estão altamente correlacionadas entre si e apresentam mensuração estável e confiável do mesmo construto^(19)^. Além disso, sugere que os participantes compreenderam bem os itens. Na versão original da escala, bem como na versão turca, o alfa de Cronbach foi de 0,88 e 0,70, respectivamente^(7,24)^.

Outro aspecto relevante diz respeito aos participantes com menos de cinco anos de formação, os quais apresentaram, em média, uma pontuação de autoeficácia 24% inferior àquela observada entre os profissionais com maior tempo de atuação. Contrariamente, estudo que investigou o nível de conhecimento de professores da educação básica sobre a asma constatou que os docentes com menor tempo de atuação obtiveram maiores escores de conhecimento^(33)^. Embora se tratem de construtos distintos, a comparação se justifica, uma vez que o conhecimento pode ser considerado um fator potencialmente associado à autoconfiança e à percepção de segurança no manejo de situações de saúde em ambiente escolar.

Por outro lado, a variável idade não apresentou diferença estatisticamente significativa. Em contraste, estudo sobre o conhecimento e a postura de professores em relação ao diabetes infantil, outra condição crônica, revelou que docentes com mais de 45 anos demonstraram maior compreensão no manejo da doença, resultado atribuído à experiência acumulada ao longo dos anos de prática profissional^(34)^.

### Limitações do estudo

Embora os resultados deste estudo encontrem respaldo na literatura, é válido apontar suas limitações. A primeira se refere ao número de participantes que pode não ser representativo de todos os professores brasileiros, especialmente considerando as variações regionais, culturais e de infraestrutura educacional. Também foi considerada uma limitação a não realização de análise da validade de critério concorrente, uma vez que não foram encontrados instrumentos que avaliassem o mesmo fenômeno. Tais limitações não comprometem a relevância do estudo, mas oferecem oportunidades para melhorias em estudos futuros ou etapas adicionais de validação.

### Contribuições para a área da enfermagem

Esta escala contribui com a área da enfermagem, sobretudo porque pode ser utilizada como uma ferramenta de análise situacional capaz de subsidiar o desenvolvimento de programas de capacitação e atualizações voltadas aos professores, contribuindo para o fortalecimento da integração entre saúde e educação.

## CONCLUSÃO

A versão em português do Brasil da *Teachers’ Self-Efficacy in Asthma Management* apresentou característica unidimensional, cargas fatoriais satisfatórias e bons níveis de confiabilidade, os quais apontam para um instrumento com evidências de estrutura interna consistente e confiável para a mensuração do construto desejado.

## Data Availability

Os dados de pesquisa disponíveis estão no corpo do artigo.
